# Safety and Drug–Drug Interaction Burden of Direct‐Acting Antiviral Therapy for Hepatitis C: A Single‐Center Community Hospital Analysis

**DOI:** 10.1155/ijh/5499912

**Published:** 2026-05-04

**Authors:** Satoru Okabe, Akira Doi, Kengo Matsumoto, Masashi Yamamoto, Koji Fukui, Tsutomu Nishida

**Affiliations:** ^1^ Department of Gastroenterology, Toyonaka Municipal Hospital, Toyonaka, Osaka, Japan, chp.toyonaka.osaka.jp

**Keywords:** direct-acting antivirals, drug–drug interactions, hepatitis C, polypharmacy

## Abstract

**Background:**

Polypharmacy leads to drug–drug interactions (DDIs) with direct‐acting antivirals (DAAs). We quantified the DDI burden (Liverpool categories) and evaluated its association with effectiveness and safety. We assessed renal function as a predictor of adverse events (AEs) and reported loss to follow‐up (LTFU).

**Methods:**

We retrospectively analyzed 145 adults with chronic hepatitis C treated with glecaprevir/pibrentasvir (GLE/PIB) or sofosbuvir/velpatasvir (SOF/VEL) between February 2018 and October 2024. The primary endpoint was sustained virological response 12 weeks after the end of treatment (SVR12); when SVR12 was missing, SVR24 was substituted (hierarchical SVR). Concomitant drugs were screened using the Liverpool HEP Drug Interaction Checker. The predictors of AEs were assessed using multivariable logistic regression and receiver operating characteristic analysis. A conservative intention‐to‐treat analysis included missing SVR data as a failure.

**Results:**

The median patient age was 67 years, and 23.4% of patients had cirrhosis. Polypharmacy (≥ 5 drugs) occurred in 26.9% of patients, DDIs in 50.3%, multiple DDIs in 14.5%, and contraindicated pairs in 1.4% (all GLE/PIB). AEs occurred in 16.6% of patients, were largely Grades 1–2, and began around Week 2. LTFU was 11.0%. The hierarchical SVR was 99.2%, whereas the conservative analysis was 88.3%. A Cr concentration ≥ 0.86 mg/dL independently predicted AEs (adjusted OR 3.4; 95% CI 1.24–9.2), whereas DDI burden and polypharmacy did not.

**Conclusions:**

Despite frequent DDIs, DDI burden was not independently associated with SVR or AEs. DAA therapy was highly effective and well tolerated. Renal function showed a modest association with AEs.

## 1. Introduction

Hepatitis C virus (HCV) infection remains a significant public health concern in Japan, particularly in the aging population. HCV contributes to cirrhosis, hepatocellular carcinoma, and long‐term burden, including excess liver‐related mortality, reduced quality of life, and greater healthcare utilization [1]. With the adoption of direct‐acting antivirals (DAAs), interferon‐free therapy has become the standard, and sustained virologic response (SVR) rates now typically exceed 95% [2], even in older patients and patients with cirrhosis [3, 4].

However, many elderly patients take multiple medications because of comorbidities. Polypharmacy, commonly defined as the use of five or more drugs [5], increases the risk of drug–drug interactions (DDIs) during DAA therapy [6, 7]. Real‐world Japanese studies have reported a high prevalence of comorbidities and a substantial DDI burden among patients with HCV, highlighting the importance of structured interaction management [1] [2].

In clinical practice, medication reconciliation and the use of validated interaction resources are recommended. Consistent with the European Association for the Study of the Liver (EASL) guidelines [8], we conducted a standardized medication review before treatment and used the Liverpool HEP resource checker, which classifies drugs into four categories: red, amber, yellow, and green [9]. However, most prior studies have relied on claims or prescription data [1, 2, 6], and few have examined patient‐level outcomes such as adverse events (AEs), treatment discontinuations, or SVR. Loss to follow‐up (LTFU) remains a challenge. When AEs lead to dropout, safety may be underestimated, and the SVR may be overestimated.

In this study, we analyzed real‐world data from a single Japanese hospital to evaluate the burden of DDIs and their relationship with safety (AEs) and effectiveness (SVR12) in patients who were treated with either glecaprevir/pibrentasvir (GLE/PIB) or sofosbuvir/velpatasvir (SOF/VEL). We also assessed polypharmacy, accounted for LTFU, and used both evaluable‐set and conservative intention‐to‐treat analyses (counting missing SVR as failure).

## 2. Methods

### 2.1. Study Design and Setting

This single‐center, retrospective, observational study was conducted at Toyonaka Municipal Hospital. We identified consecutive adults with HCV infection who began treatment with GLE/PIB or SOF/VEL between February 2018 and October 2024. The data from electronic medical records included patient demographics, liver disease stage, laboratory values, treatment course, AEs, and concomitant medications.

### 2.2. Virologic Assessment

HCV RNA levels were measured using a validated real‐time PCR assay with a detection limit of 1.1 IU/mL. “Undetectable” was defined as the target not being detected.

### 2.3. Patients, Follow‐Up, Endpoints, and Analysis Sets

Eligible patients received at least one dose of the DAA regimen at our institution. Patients with HBV/HIV coinfection or patients who initiated treatment at other institutions were excluded. Follow‐up was extended from treatment initiation to posttreatment assessment.

The primary endpoint was a SVR at 12 ± 2 weeks after the end of treatment (EOT) (SVR12). Because some patients missed the SVR12 window but returned later, we predefined a hierarchical SVR outcome: SVR12 was prioritized; if unavailable, SVR24 (24 ± 4 weeks after treatment) was used. This approach is supported by data demonstrating high concordance between SVR12 and SVR24, with late relapse being rare [8, 10, 11]. HCV RNA negativity at the EOT was not considered an SVR.

EOT was defined as the final day of the last prescribed supply for the planned treatment duration. For example, in an 8‐week course with a 2‐week refill at Week 6, Day 56 was considered the EOT. If early discontinuation was documented, the last dose was used.

### 2.4. Analysis Sets

Analysis sets were as follows:

• SVR12‐evaluable set: patients whose SVR12 or SVR24 data were included.
• All‐treated (safety) set: All patients receiving at least one dose; used for AE, treatment completion, and LTFU analyses.
• Missing‐as‐failure analysis: A conservative intention‐to‐treat approach in which all patients in the all‐treated set were included and missing SVR assessments were counted as failures.

LTFU was defined as the absence of SVR and SVR24 data owing to factors such as relocation, transfer, patient preference, or loss of contact. Patients with SVR12 but not SVR24 were not considered LTFU; patients with missing SVR12 but available SVR24 data were considered SVR positive.

### 2.5. Laboratory Definitions

Serum creatinine levels were measured using an IDMS‐traceable enzymatic method. The estimated glomerular filtration rate (eGFR) was calculated using the Japanese Society of Nephrology equation (JSN 2012) [12]. Baseline values were defined as values obtained on the day of DAA initiation or at the most recent pretreatment visit before DAA initiation.

### 2.6. DDI Assessment

All concomitant prescription drugs, both regular and as needed, were reviewed using the Liverpool HEP Drug Interaction Checker [9]. DDIs were classified into four categories:

• Red: do not coadminister
• Amber: potential interaction
• Yellow: weak interaction
• Green: no interaction expected

The highest interaction level observed during treatment was recorded for every patient. Multiple DDIs were defined as two or more drugs with yellow or higher interaction levels. Over‐the‐counter medications and supplements were not systematically collected, which is a limitation of this study.

Importantly, during most of our study period (2018–2024), contemporaneous guidance discouraged coadministration of strong enzyme inducers with DAAs. In the current Liverpool HEP Drug Interaction Checker entry for GLE/PIB with carbamazepine, the header reads “potential interaction,” but the summary clearly states “Coadministration is not recommended [13],” and the current manufacturer labeling for MAVYRET (GLE/PIB) [14] and EPCLUSA (SOF/VEL) [15] likewise advises against use with carbamazepine because this drug reduces antiviral exposure. Accordingly, we classified carbamazepine as red (do not coadminister) throughout and applied the same approach to inducers with analogous mechanisms to reflect prevailing clinical practice [8].

### 2.7. AEs

AEs were abstracted from clinical records and classified according to type, severity, timing, and treatment discontinuation. The severity was graded according to the Common Terminology Criteria for Adverse Events (CTCAE) v5.0 [16]. The candidate risk factors included age, sex, liver disease stage, DAA regimen, DDI characteristics (presence, level, and number), polypharmacy, and renal function (creatinine level and eGFR).

### 2.8. Effectiveness (SVR12)

SVR12 was defined as an undetectable level of HCV RNA at 12 ± 2 weeks after EOT. The primary analysis used an evaluable set. A conservative intention‐to‐treat analysis was performed, with missing SVR data treated as failures.

### 2.9. Polypharmacy and Age Stratification

In accordance with the WHO guidelines, polypharmacy was defined as the concurrent use of five or more medications [5]. The age groups were < 75 and ≥ 75 years, on the basis of the criteria of Japan′s Late‐Stage Elderly Healthcare System [17].

### 2.10. Visualization of Patient Flow and LTFU

Patient characteristics, including treatment completion, SVR12 assessment, and LTFU, are illustrated in a flow chart. The reasons for LTFU are shown at the terminal nodes.

### 2.11. Ethics

This study was approved by the Institutional Review Board of Toyonaka Municipal Hospital (Approval No. 2024‐11‐03) and was conducted in accordance with the Declaration of Helsinki and local regulations. Because of the retrospective design, the need for written informed consent was waived, and an opt‐out procedure was implemented via the institutional website and on‐site notice boards. All the data were anonymized prior to the analysis.

### 2.12. Statistical Analysis

Continuous variables are summarized as medians and interquartile ranges (IQRs), and categorical variables are summarized as counts and percentages. The Mann–Whitney *U* test was used for continuous variables, and Fisher′s exact test or the *χ* ^2^ test was used for categorical comparisons. For the prediction of AEs, multivariable logistic regression was conducted, including age as a continuous variable by default. Variables with *p* < 0.10 in the univariable analysis or with clinical relevance were included. Multicollinearity was assessed using the variance inflation factor (VIF). ROC curve analysis was used to evaluate the predictive value of creatinine levels and the eGFR, with cutoff values determined using the Youden index. All analyses were performed using JMP Pro Version 17.2 (SAS Institute Inc., Cary, North Carolina, United States), and statistical significance was set at *p* < 0.05.

## 3. Results

### 3.1. Patient Characteristics

**Figure 1:**
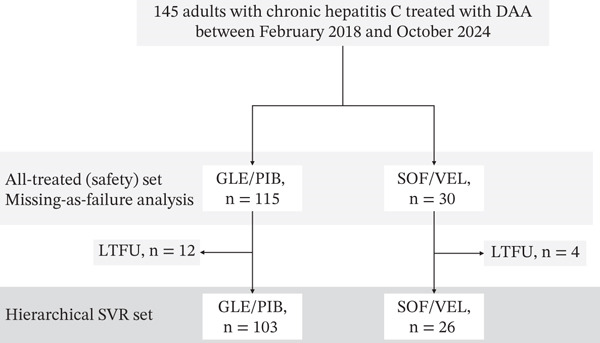
Patient flow diagram. Study cohort and analysis populations. The all‐treated (safety) set included all patients who initiated DAA therapy. SVR was assessed in an evaluable set; in addition, a conservative intention‐to‐treat analysis assuming that missing SVR = failure was performed. Loss to follow‐up (LTFU) at posttreatment assessment is shown. No treatment‐related deaths were observed. Abbreviations: GLE/PIB, glecaprevir/pibrentasvir; SOF/VEL, sofosbuvir/velpatasvir; SVR, sustained virologic response; ITT, intention‐to‐treat; LTFU, loss to follow‐up.

A total of 145 patients were included, of whom 115 received GLE/PIB, and 30 received SOF/VEL (Figure 1). The median patient age was 67 years (range, 30–90 years), and 72 (49.7%) were male. Most of the patients were treatment‐naïve (89.7%). The liver disease stages included chronic hepatitis (76.6%), compensated cirrhosis (12.4%), and decompensated cirrhosis (11.0%). The HCV genotypes were 1 (*n* = 89), 2 (*n* = 54), and 3 (*n* = 2) (Table 1).

**Table 1:** Patient background.

	All	GLE/PIB	SOF/VEL	*p*value
Patient number, *n*	145	115	30	
Age (years), median (IQR)	67 (54–77)	66 (53–75)	76.5 (61.8–82.37)	0.005
Sex: male, *n*, (%)	72 (50)	58 (50)	14 (47)	0.838
Prior HCV treatment: yes, *n*, (%)	15 (10.3)	12 (10.4)	3 (10)	0.146
HCV genotype,1/2/3, *n*	89/54/2	69/45/1	20/9/1	0.416
HCV‐RNA (Log IU/mL), median (IQR)	6.3 (5.8–6.7)	6.4 (5.6–6.7)	6.2 (5.8–6.8)	0.828
Liver disease stage: CH/c‐LC/de‐LC	111/18/16	100/15/0	11/3/16	< 0.0001
History of HCC treatment, yes, *n*, (%)	11 (7.6)	4 (3.5)	7 (23)	0.002
Number of concomitant drugs, 0/1–4/5–, *n*, (5–, %)	36/70/39 (27)	34/55/26 (23)	2/15/13 (43)	0.019
Platelet count (×10^4^/*μ*L), median (IQR)	19.7 (13.9–22.8)	20.2 (16.7–23.3)	11.1 (7.1–18.7)	< 0.0001
Total bilirubin (mg/dL), median (IQR)	0.69 (0.52–0.95)	0.66 (0.51–0.84)	0.97 (0.75–1.3)	0.0001
AST (IU/L), median (IQR)	36 (26–56.5)	33 (25–54)	55 (47–69)	0.0002
ALT (IU/L), median (IQR)	38 (22.5–55.5)	37 (21–58)	47 (29.5–53.3)	0.226
Creatinine (mg/dL), median (IQR)	0.78 (0.64–0.98)	0.77 (0.63–1)	0.89 (0.66–0.97)	0.414
eGFR (mL/min/1.73m^2^), median (IQR)	67 (53.9–78.5)	68.3 (56.5–78.6)	57.6 (47.1–73.7)	0.104
Albumin (g/dL), median (IQR)	4.0 (3.6–4.3)	4.1 (3.8–4.3)	3.3 (3.1–3.6)	< 0.0001
Prothrombin time (%), median (IQR)	100 (88–113)	100 (92–111)	95.5 (79–118)	0.267

Abbreviations: ALT, alanine aminotransferase; AST, aspartate aminotransferase; CH, chronic hepatitis; c‐LC, compensated liver cirrhosis; de‐LC, decompensated liver cirrhosis; eGFR, estimated glomerular filtration rate; GLE/PIB, glecaprevir/pibrentasvir; HCC, hepatocellular carcinoma; HCV, hepatitis C virus; HCV‐RNA, hepatitis C virus RNA; IQR, interquartile range; *n*, number; SOF/VEL: sofosbuvir/velpatasvir.

At baseline, patients were taking a median of three concomitant medications (IQR 1–6), and polypharmacy (≥ 5 drugs) was observed in 26.9% of patients. Compared with those in the GLE/PIB group, patients in the SOF/VEL group were older (median age, 76.5 vs. 66.0 years) and had lower serum albumin levels (3.3 vs. 4.1 g/dL) and lower eGFRs (57.7 vs. 68.3 mL/min/1.73 m^2^) (Table 1).

### 3.2. Treatment Effectiveness (SVR12)

Among the 145 patients, 129 had posttreatment SVR data. When the hierarchical SVR endpoint (SVR12 prioritized, SVR24 substituted when missing) was used, 128/129 (99.2%) patients achieved SVR. Virologic failure occurred in one patient in the SOF/VEL group. In the conservative intent‐to‐treat analysis, in which a lack of SVR data was counted as failure, the overall SVR rate was 88.3% (128/145 patients). All patients who discontinued treatment because of AEs achieved SVR. No treatment‐related deaths were observed.

### 3.3. AEs

**Figure 2:**
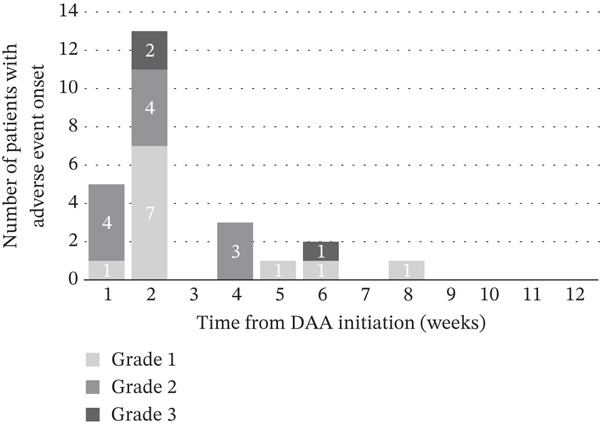
Time to onset of adverse events during treatment with direct‐acting antivirals (DAAs). Bars indicate the number of patients with adverse event onset in each week after initiation of DAAs, stratified by CTCAE v5.0 grade (Grades 1, 2, and 3). Most adverse events occurred within the first 2 weeks of treatment, including one event at Week 8. Abbreviations: DAA, direct‐acting antiviral; AE, adverse event; CTCAE, Common Terminology Criteria for Adverse Events.

AEs occurred in 24 patients (16.6%): Grade 1 (*n* = 17), Grade 2 (*n* = 5), and Grade 3 (*n* = 2); no Grade 4 or 5 events occurred. The most common AEs were pruritus (5.5%), fatigue (4.1%), and elevated bilirubin levels (2.1%) (Table 2). The median onset time was Week 2 (IQR 2–2), and most events were mild (Figure 2). The incidence of AEs was similar between the regimens: 16.5% for GLE/PIB and 16.7% for SOF/VEL. Two patients discontinued treatment owing to AEs but still achieved SVR. Patient‐level details are presented in Table S1.

**Table 2:** Adverse events.

Adverse events (AEs)	ALL, *n* = 145		GLE/PIB, *n* = 115		SOF/VEL, *n* = 30	
	*n*	Gr 1/2/3	*n*	Gr 1/2/3	*n*	Gr 1/2/3
Number of patients with AEs, *n* (%)	24 (16.6)	17/5/2	19 (16.5)	14/3/2	5 (16.6)	3/2/0
Total number of AEs, *n* (%)	28 (19.3)	21/5/2	23 (20.0)	18/3/2	5 (16.6)	3/2/0
Pruritus, *n* (%)	8 (5.5)	8/0/0	6 (5.2)	6/0/0	2 (6.7)	2/0/0
Malaise, *n* (%)	6 (4.1)	6/0/0	5 (4.4)	5/0/0	1 (3.3)	1/0/0
Blood bilirubin increased, *n* (%)	3 (2.1)	0/2/1	3 (2.6)	0/2/1	0 (0)	0/0/0
Edema, *n* (%)	2 (1.4)	0/2/0	1 (0.7)	0/1/0	1 (3.3)	0/1/0
Anemia, *n* (%)	2 (1.4)	0/1/1	1 (0.7)	0/0/1	1 (3.3)	0/1/0
Nausea, *n* (%)	1 (0.7)	1/0/0	1 (0.7)	1/0/0	0 (0)	0/0/0
Rash, *n* (%)	1 (0.7)	1/0/0	1 (0.7)	1/0/0	0 (0)	0/0/0
ALT elevation, *n* (%)	1 (0.7)	1/0/0	1 (0.7)	1/0/0	0 (0)	0/0/0
Diarrhea, *n* (%)	1 (0.7)	1/0/0	1 (0.7)	1/0/0	0 (0)	0/0/0
Headache, *n* (%)	1 (0.7)	1/0/0	1 (0.7)	1/0/0	0 (0)	0/0/0
Cough, *n* (%)	1 (0.7)	1/0/0	1 (0.7)	1/0/0	0 (0)	0/0/0
Drowsiness, *n* (%)	1 (0.7)	1/0/0	1 (0.7)	1/0/0	0 (0)	0/0/0

Abbreviations: AEs, adverse events; ALT, alanine aminotransferase; GLE/PIB, glecaprevir/pibrentasvir; Gr, grade; *n*, number; SOF/VEL: sofosbuvir/velpatasvir.

The baseline characteristics of patients with and without AEs are shown in Table 3. Patients with AEs had higher baseline serum creatinine levels (median 0.97 vs. 0.77 mg/dL, *p* = 0.017) and a different distribution of liver disease stages (*p* = 0.039). There were trends toward higher total bilirubin levels (*p* = 0.058) and lower eGFRs (*p* = 0.079). Polypharmacy (33.3% vs. 25.6%, *p* = 0.718) and DDI metrics (presence, severity, or multiple DDIs) did not differ significantly (Table 3).

**Table 3:** Baseline characteristics stratified by the occurrence of adverse events (AEs) during DAA therapy.

	Any AEs, *n* = 24	Without AEs, *n* = 121	*p* value
Age (years), median (IQR)	69.5(62–78.5)	67(53.5–77)	0.511
Sex: male, *n*, (%)	15(62.5)	57(47.1)	0.189
DAA (GLE/PIB, SOF/VEL)	19 (GLE/PIB), 5 (SOF/VEL)	96 (GLE/PIB), 25 (SOF/VEL)	1
DAA treatment: nonnaïve, *n*, (%)	5 (20.8)	10 (8.2)	0.076
HCV genotype 1/2/3, *n*	16/8/0	73/46/2	0.724
HCV‐RNA (Log IU/mL), median (IQR)	6.5 (6–6.8)	6.3 (5.6–6.7)	0.482
Liver disease stage: CH/c‐LC/de‐LC	14/5/5	111/18/16	0.039
History of HCC treatment: Yes, *n*, (%)	3 (12.5)	8 (6.6)	0.392
Platelets (×10^4^/*μ*L), median (IQR)	18.2 (6.5–45.5)	19.9 (3.4–36.8)	0.33
Total bilirubin (mg/dL), median (IQR)	0.8 (0.6–1.1)	0.7 (0.5–0.9)	0.058
AST (IU/L), median (IQR)	33 (26–54)	39 (26–58)	0.400
ALT (IU/L), median (IQR)	37 (19–43)	41 (23–61)	0.096
Creatinine (mg/dL), median (IQR)	0.97 (0.68–1.15)	0.77 (0.63–0.93)	0.017
eGFR (mL/min/1.73m^2^), median (IQR)	58.9 (37.1–79.4)	67.6 (56.1–78.5)	0.079
Albumin (g/dL), median (IQR)	3.7 (3.4–4.1)	4.0 (3.6–4.3)	0.128
Prothrombin time (%), median (IQR)	95 (83–102)	101 (92–115)	0.029
No. of comedications	4 (0–11)	3 (0–13)	0.272
No. of comedications, 0/1–4/5–, *n*, (5–, %)	5/11/8 (33.3)	31/59/31 (25.6)	0.718
DDI patients, *n*, (%)	14 (58)	59 (49)	0.601
Multi‐DDI^a^ patients, *n*, (%)	6 (25)	15 (12.3)	0.119
DDI class (green, yellow, amber, red), *n*, (%)	10/2/11/1(42/8/46/4)	62/21/37/1(51/17/31/1)	0.219

Abbreviations: AEs, adverse events; ALT, alanine aminotransferase; CH, chronic hepatitis; c‐LC, compensated liver cirrhosis; DAA, direct‐acting antiviral; DDI, drug–drug interaction; de‐LC, decompensated liver cirrhosis; eGFR, estimated glomerular filtration rate; GLE/PIB, glecaprevir/pibrentasvir; HCC, hepatocellular carcinoma; HCV, hepatitis C virus; HCV‐RNA, hepatitis C virus RNA; multi‐DDI, multiple drug–drug interactions; *n*, number; SOF/VEL, sofosbuvir/velpatasvir.

^a^Multiple DDIs were defined as ≥ 2 concomitant drugs yielding yellow/amber/red per the Liverpool HEP Drug Interaction Checker (highest category per patient shown). Over‐the‐counter products and supplements were not included.

### 3.4. DDIs

**Figure 3:**
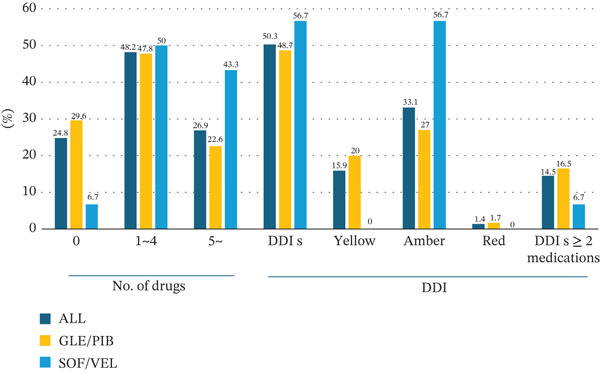
Concomitant medication burden and Liverpool interaction categories during DAA therapy. (A) Distribution of the number of concomitant drugs per patient (0, 1–4, ≥ 5). (B) Distribution of Liverpool HEP Drug Interaction categories (green, yellow, amber, and red) summarized at the patient level during treatment. “Multiple DDIs” was defined as ≥ 2 concomitant drugs categorized as yellow or higher. Abbreviations: DAA, direct‐acting antiviral; DDI, drug–drug interaction.

Overall, 50.3% of patients had at least one DDI, and 14.5% had multiple DDIs (≥ 2 drugs, yellow or higher). Contraindicated (red) interactions occurred in 2 (1.4%) patients, both of whom were administered GLE/PIB. The prevalence of DDI was 48.7% in the GLE/PIB group and 56.7% in the SOF/VEL group (Figure 3). The most common classes of interacting drugs were proton‐pump inhibitors (20.7%) and statins (4.1%). DDI metrics were not significantly associated with the occurrence of AEs. The DDI distribution is shown in Table 4.

**Table 4:** Liverpool category‐based drug–drug interaction profile during DAA therapy (patient‐ and drug‐level summary).

Concomitant drug class	Drug name	Overall, *n*	GLE/PIB, *n*	Liverpool category (GLE/PIB)	SOF/VEL, *n*	Liverpool category (SOF/VEL)
All patients		145	115		30	
Antibacterials	Clarithromycin	1	1	Weak	0	None
	Erythromycin	1	1	Potential	0	None
Cardiovascular	Digoxin	1	1	Potential	0	Potential
	Candesartan cilexetil	9	9	Weak	0	None
	Irbesartan	3	3	Potential	0	None
	Olmesartan medoxomil	5	5	Potential	0	None
	Telmisartan	5	5	Potential	0	None
	Carvedilol	7	6	Potential	1	Potential
Anticonvulsants	Carbamazepine	1	1	Contraindication		
Antipsychotics/neuroleptics	Quetiapine fumarate	1	1	Potential		
	Risperidone	2	2	Weak		
Gastrointestinal agents	Famotidine	6	4	Weak	2	Potential
	Esomeprazole magnesium hydrate	13	3	Weak	10	Potential
	Lansoprazole	9	8	Weak	1	Potential
	Omeprazole	1	1	Weak	0	Potential
	Rabeprazole sodium	5	3	Weak	2	Potential
	Vonoprazan fumarate	9	7	Weak	2	Potential
	Domperidone	2	2	Potential	0	None
Lipid‐lowering agents	Atorvastatin calcium hydrate	1	1	Contraindication	0	Potential
	Ezetimibe	2	2	Potential	0	None
	Pitavastatin calcium hydrate	2	2	Potential	0	Potential
	Rosuvastatin calcium	3	3	Potential	0	None
Anticoagulant/antiplatelet and fibrinolytic	Apixaban	2	1	Potential	1	Potential
	Edoxaban tosilate hydrate	1	0	Potential	1	Potential
	Rivaroxaban	2	2	Potential	0	None
Oral antidiabetic agent	Repaglinide	2	2	Potential	0	None
Antiallergic agent	Fexofenadine hydrochloride	1	1	Weak	0	None
Others	Etizolam	3	3	Potential	0	None
	Mirabegron	1	1	Potential	0	None

*Note:* None: no interaction expected; weak: weak interaction; potential: potential clinically significant interaction; contraindication: do not coadminister.

Abbreviations: DAA, direct‐acting antiviral; GLE/PIB, glecaprevir/pibrentasvir; *n*, number; SOF/VEL, sofosbuvir/velpatasvir.

### 3.5. Factors Associated With AEs

ROC analysis revealed a creatinine cutoff of 0.86 mg/dL (AUC = 0.655; sensitivity, 70.8%; specificity, 62.8%). According to the results of the univariable analyses, a creatinine concentration ≥ 0.86 mg/dL was significantly associated with AEs (OR 4.1, 95% CI 1.58–10.7; *p* = 0.0037), and the incidence of decompensated cirrhosis showed a nonsignificant trend (OR 3.15; *p* = 0.060). In the multivariable logistic regression analysis for age, a creatinine concentration ≥ 0.86 mg/dL remained an independent predictor (adjusted OR 3.4; 95% CI 1.24–9.2; *p* = 0.017). Neither DDI burden nor polypharmacy was independently associated with AEs (Table 5).

**Table 5:** Factors associated with adverse events during DAA therapy: univariable and age‐adjusted multivariable logistic regression.

		Univariate			Multivariate^a^			VIF
	Reference	OR	95% CI	*p*value	OR	95% CI	*p* value	
< 65 years	≥ 65 years	0.62	0.25–1.56	0.3098				
Female	Male	0.53	0.22–1.34	0.1724				
Liver disease stage	CH							
De‐LC		3.15	0.95–10.4	0.0602	2.54	0.69–9.4	0.1622	1.08
C‐LC		2.67	0.82–8.6	0.1017	2.45	0.71–8.5	0.1554	1.03
GLE/PIB	SOF/VEL	0.99	0.33–2.91	0.9848				
Past DAA treatment history	Naïve	2.92	0.90–9.5	0.0747	2.04	0.58–7.3	0.2692	1.05
The number of drugs	0							
1‐4		1.16	0.36–3.63	0.8038				
5‐		1.60	0.47–5.4	0.4514				
DDI, yes	No	1.47	0.61–3.57	0.3933				
Weak DDI, present	No	0.59	0.12–2.92	0.5179				
Potential DDI, present	No	1.84	0.71–4.76	0.2062				
Contraindication DDI, present	No	6.2	0.36–107	0.2098				
Cre ≥ 0.86 mg/dL	Cre < 0.86	4.1	1.58–10.7	0.0037	3.4	1.24‐9.2	0.0172	1.09

*Note:* The VIF values for the variables included in the multivariable model ranged from 1.03 to 1.09, indicating no substantial multicollinearity. Naïve: treatment‐naïve, weak: weak interaction, potential: potential clinically significant interaction, contraindication: do not coadminister, Cre: serum creatinine.

Abbreviations: 95% CI, 95% confidence interval; CH, chronic hepatitis; C‐LC, compensated liver cirrhosis; DAA, direct‐acting antiviral; DDI, drug–drug interaction; De‐LC, decompensated liver cirrhosis; GLE/PIB, glecaprevir/pibrentasvir; OR, odds ratio; SOF/VEL, sofosbuvir/velpatasvir; VIF, variance inflation factor.

^a^Adjusted by age as a continuous variable.

### 3.6. LTFU

Sixteen patients (11.0%) were LTFU and lacked both SVR12 and SVR24 assessments (Table S2). LTFU occurred in 10.4% (GLE/PIB) and 13.3% (SOF/VEL) of the patients (Figure 1). Most patients with LTFU had chronic hepatitis and were male (75%), with a median age of 60 years. In 81% of the cases, the last contact occurred at the EOT. Only one patient discontinued treatment early. Mild AEs were documented in 25.0% of the LTFU patients. Half of the patients had at least one DDI, and 37.5% met the polypharmacy threshold (Table S2).

## 4. Discussion

Our main finding was that although DDIs were common in this polypharmacy cohort, DDI burden was not independently associated with treatment effectiveness or safety. These findings suggest that structured pretreatment medication reviews and appropriate DAA selection can mitigate the risk of DDIs in routine care. The secondary finding was that baseline renal function, particularly the serum creatinine level, was independently associated with AEs.

Our findings align with prior work regarding overall effectiveness and tolerability, but they differ in terms of regimen‐specific AE rates. A Spanish EMR study (BIG‐PAC) using the Liverpool checker revealed that AEs were more frequent among patients with multiple DDIs and were reported more often with GLE/PIB than with SOF/VEL (18.3% vs. 5.8%) [18]. However, the BIG‐PAC study lacked SVR data and relied on administrative records. In contrast, our cohort analysis included direct SVR12 measurements and formal AE grading, and we found the incidence of AEs to be comparable between GLE/PIB (16.5%) and SOF/VEL (16.6%). The high effectiveness and low AE rates in our study are consistent with the findings of Japanese clinical trials [19, 20]. Real‐world evidence from Japan also revealed a 99% SVR12 rate and good tolerability of GLE/PIB [21]. Taken together, these data reinforce that DAA therapy remains effective and well tolerated, even in settings with potential DDIs and polypharmacy, when appropriate risk management is in place.

In the multivariate analysis, elevated baseline serum creatinine concentration (≥0.86 mg/dL) was an independent predictor of AEs. Although DAAs are primarily metabolized hepatically, renal function may reflect broader vulnerability, including frailty, inflammation, and reduced clearance of comedications. These findings are clinically relevant even for regimens such as GLE/PIB, which are safe for chronic kidney disease, and SOF/VEL, which includes a renally cleared component. Most AEs occurred within 2 weeks of treatment initiation, which supports early monitoring, particularly in patients with impaired renal function.

LTFU occurred in 11% of patients, mostly after the completion of treatment. Only one patient discontinued treatment early because of a Grade 3 AE. Our LTFU rate is comparable with that in previous Japanese real‐world studies, such as that of Kuwano et al., who reported an 8.4% LTFU rate before SVR12 and reported that younger age was a risk factor [22]. Similarly, German data indicated an 8% prevalence of LTFU [23]. Although overall posttreatment follow‐up remains high in Japan, younger patients are more likely to discontinue their visits [24]. In our cohort, the patients who were LTFU were slightly younger and predominantly male. Most patients appeared to have completed therapy, suggesting that missed SVR testing, rather than failure during treatment, was the issue. This supports the use of pragmatic interventions, such as reminders or flexible scheduling, around the SVR window.

This study had several limitations. This was a single‐center retrospective analysis with a limited number of AEs, which reduced the statistical power. DDI classification was based on interaction checkers, not serum drug levels, and may have overestimated or underestimated clinical relevance. Mild or unreported AEs may have been missed because of the reliance on medical records. Adherence was not directly assessed. Finally, LTFU may introduce bias, although we addressed this problem by performing both evaluable‐set and conservative ITT analyses.

In this real‐world cohort, DAAs achieved high SVR with good tolerability despite frequent potential DDIs, provided that standardized medication review and validated interaction tools were used. Renal impairment emerged as a signal to consider closer monitoring and regimen selection in specific contexts, but not as a barrier to effectiveness. To optimize safety and reduce AE‐related nonadherence, treatment discontinuation, and LTFU, prospective multicenter work should operationalize risk‐based pathways, focusing on impaired renal function, high‐risk comedication clusters such as acid suppressants, statins, or cardiovascular drugs that may warrant closer monitoring and structured DDI screening and early AE check‐ins.

## Author Contributions

Study concept and design: Satoru Okabe, Kengo Matsumoto, and Tsutomu Nishida; drafting of the manuscript: Satoru Okabe and Tsutomu Nishida; data acquisition: Satoru Okabe; data analysis and interpretation: Satoru Okabe, Akira Doi, Kengo Matsumoto, and Tsutomu Nishida; critical revision of the manuscript for important intellectual content: Satoru Okabe, Akira Doi, Kengo Matsumoto, Masashi Yamamoto, Koji Fukui, and Tsutomu Nishida; study supervision: Akira Doi and Kengo Matsumoto.

## Funding

No funding was received for this manuscript.

## Disclosure

All authors gave final approval of the manuscript.

## Ethics Statement

This study was approved by the Institutional Review Board of Toyonaka Municipal Hospital (Approval No. 2024‐11‐03).

## Conflicts of Interest

The authors declare no conflicts of interest.

## Supporting information


**Supporting Information** Additional supporting information can be found online in the Supporting Information section. Table S1: Patient‐level details of adverse events (CTCAE v5.0 classification). Table S2: Loss to follow‐up (LTFU): reasons and timing of last contact in patients without SVR12 or SVR24 assessment.

## Data Availability

The data that support the findings of this study are available on request from the corresponding author. The data are not publicly available due to privacy or ethical restrictions.
