# Supplementary material for: Safety and Drug–Drug Interaction Burden of Direct‐Acting Antiviral Therapy for Hepatitis C: A Single‐Center Community Hospital Analysis
Source: Int J Hepatol. 2026 May 4;2026:5499912. doi: 10.1155/ijh/5499912 (PMC13136848; doi:10.1155/ijh/5499912)
Supplement: Supplementary file 1 — Supporting Information Additional supporting information can be found online in the Supporting Information section. Table S1: Patient‐level details of adverse events (CTCAE v5.0 classification). Table S2: Loss to follow‐up (LTFU): reasons and timing of last contact in patients without SVR12 or SVR24 assessment. [file IJH-2026-5499912-s001.docx]

Supplementary Table 1. Patient-level details of adverse events (CTCAE v5.0 classification)

| Age | Sex | Genotype | CH/c-LC/de-LC | History of HCC treatment | History of DAA treatment | DAA | Duration of administration | No. of medications | DDI | AE | CTCAE Grade | Time of onset of adverse events |
| --- | --- | --- | --- | --- | --- | --- | --- | --- | --- | --- | --- | --- |
| 39 | F | 2 | CH | No | Naïve | GLE/PIB | 8 | 0 | No | Pruritus | 1 | 4 w |
| 72 | F | 1 | CH | No | Naïve | GLE/PIB | 8 | 1 | No | Rash | 1 | 5 w |
| 66 | M | 1 | CH | No | Naïve | GLE/PIB | 8 | 4 | Potential: 1 drug | Malaise | 1 | 2 w |
| 73 | M | 1 | CH | No | Naïve | GLE/PIB | 8 | 4 | Weak:  1 drug | Malaise, pruritus | 1 | 1 w |
| 71 | F | 1 | CH | No | Naïve | GLE/PIB | 8 | 2 | Potential: 1 drug | Malaise, pruritus | 1 | 1 w |
| 51 | M | 2 | CH | No | Naïve | GLE/PIB | 8 | 0 | No | Nausea | 1 | 1 w |
| 65 | F | 1 | CH | No | Naïve | GLE/PIB | 8 | 2 | No | Malaise | 1 | 2 w |
| 66 | M | 1 | c-LC | No | Naïve | GLE/PIB | 12 | 0 | No | Malaise, pruritus | 1 | 2 w |
| 54 | M | 2 | CH | No | Yes | GLE/PIB | 8 | 0 | No | Headache | 1 | 2 w |
| 62 | M | 1 | c-LC | No | Naïve | GLE/PIB | 12 | 8 | No | Pruritus,  drowsiness | 1 | 2 w |
| 77 | M | 1 | CH | No | Yes | GLE/PIB | 8 | 10 | Potential, weak: each 1 drug | ALT elevation | 1 | 2 w |
| 42 | M | 1 | CH | No | Naïve | GLE/PIB | 8 | 7 | Contraindication, potential: each 1 drug | Diarrhea | 1 | 6 w |
| 70 | M | 2 | CH | No | Naïve | GLE/PIB | 8 | 6 | Potential, weak: each 1 drug | Cough | 1 | 1 w |
| 55 | F | 1 | CH | No | Naïve | GLE/PIB | 8 | 0 | No | Pruritus | 1 | 2 w |
| 84 | M | 1 | de-LC | No | Naïve | SOF/VEL | 12 | 5 | Potential: 1 drug | Malaise | 1 | 8 w |
| 62 | M | 1 | de-LC | No | Naïve | SOF/VEL | 12 | 6 | Potential: 1 drug | Pruritus | 1 | 4 w |
| 62 | M | 1 | de-LC | No | Naïve | SOF/VEL | 12 | 4 | No | Pruritus | 1 | 4 w |
| 69 | M | 1 | CH | No | Yes | GLE/PIB | 5 | 11 | Potential: 3 drugs | Edema | 2 | 1 w |
| 86 | M | 2 | de-LC | No | Yes | SOF/VEL | 12 | 6 | No | Edema | 2 | 2 w |
| 80 | F | 2 | c-LC | No | Naïve | GLE/PIB | 12 | 2 | Weak:  1 drug | Blood bilirubin increased | 2 | 2 w |
| 79 | F | 2 | c-LC | No | Naïve | GLE/PIB | 12 | 5 | Potential: 2 drugs,  weak: 1 drug | Blood bilirubin increased | 2 | 2 w |
| 89 | F | 2 | CH | No | Naïve | GLE/PIB | 3 | 4 | Potential: 1 drug | Blood bilirubin increased | 3 | 2 w |
| 82 | F | 1 | De-LC | Yes | Yes | SOF/VEL | 12 | 5 | Potential: 1 drug | Anemia | 2 | 2 w |
| 70 | F | 1 | c-LC | Yes | Naïve | GLE/PIB | 12 | 8 | Potential, weak: each 1 drug | Anemia | 3 | 2 w |

CTCAE: Common Terminology Criteria for Adverse Events, AE: adverse event, w: weeks F: female, M: male, CH: chronic hepatitis, c-LC: compensated liver cirrhosis, de-LC: decompensated liver cirrhosis, HCC: hepatocellular carcinoma, Naïve: treatment-naïve, DAA: direct-acting antiviral, GLE/PIB: glecaprevir/pibrentasvir, SOF/VEL: sofosbuvir/velpatasvir, No.: number, DDI: drug–drug interaction, None: no interaction expected, Weak: weak interaction, Potential: potential clinically significant interaction, Con: contraindication (do not coadminister).

Supplementary Table 2. Loss to follow-up (LTFU): reasons and timing of last contact of patients without SVR12 or SVR24 assessment

| LTFU  case | Age | Sex | Liver disease stage | DAA | Duration of administration  (weeks) | Comorbidities | Number of concomitant drugs | DDI | AE | CTCAE Grade | End of follow up |
| --- | --- | --- | --- | --- | --- | --- | --- | --- | --- | --- | --- |
| 1 | 71 | F | CH | GLE/PIB | 8 | Emaciation | 2 | Weak: 1 drug | No | 0 | EOT |
| 2 | 44 | M | CH | GLE/PIB | 8 | No | 0 | No | No | 0 | EOT |
| 3 | 42 | M | CH | GLE/PIB | 8 | Epilepsy, constipation, insomnia | 7 | Contraindication, potentially: each 1 drug | Diarrhea | 1 | 6 w |
| 4 | 70 | M | CH | GLE/PIB | 8 | Interstitial pneumonia | 6 | Potential, weak: each 1 drug | Cough | 1 | EOT |
| 5 | 55 | F | CH | GLE/PIB | 8 | No | 0 | No | Pruritus | 1 | EOT |
| 6 | 51 | M | CH | GLE/PIB | 8 | Depression | 6 | Potential: 2 drugs | No | 0 | EOT |
| 7 | 83 | M | CH | GLE/PIB | 8 | Benign prostatic hyperplasia | 4 | No | No | 0 | EOT |
| 8 | 51 | M | c-LC | GLE/PIB | 12 | Chronic renal failure, diabetes | 9 | Potential, weak: each 1 drug | No | 0 | EOT |
| 9 | 75 | M | CH | GLE/PIB | 8 | Chronic renal failure | 8 | Weak: 1 drug | No | 0 | EOT |
| 10 | 58 | M | CH | GLE/PIB | 8 | No | 0 | No | No | 0 | 6 w |
| 11 | 38 | M | CH | GLE/PIB | 8 | No | 0 | No | No | 0 | EOT |
| 12 | 89 | F | CH | GLE/PIB | 3 | Dyslipidemia, hypertension | 4 | Potential: 1 drug | Blood bilirubin increased | 3 | 4 w |
| 13 | 61 | M | CH | SOF/VEL | 12 | Diabetes | 9 | No | No | 0 | EOT |
| 14 | 63 | M | CH | SOF/VEL | 12 | Diabetes | 2 | No | No | 0 | EOT |
| 15 | 53 | M | De-LC | SOF/VEL | 12 | Chronic heart failure | 3 | Potential: 1 drug | No | 0 | EOT |
| 16 | 79 | F | De-LC | SOF/VEL | 12 | Hypertension | 1 | No | No | 0 | EOT |

LTFU: loss to follow-up, SVR: sustained virological response, SVR12/24: 12/24 weeks after the end of treatment, F: female, M: male, CH: chronic hepatitis, c-LC: compensated liver cirrhosis, de-LC: decompensated liver cirrhosis, DAA: direct-acting antiviral, GLE/PIB: glecaprevir/pibrentasvir, SOF/VEL: sofosbuvir/velpatasvir, DDI: drug–drug interaction, None: no interaction expected, Weak: weak interaction, Potential: potential clinically significant interaction, Contraindication: do not coadminister, AE: adverse event, CTCAE: Common Terminology Criteria for Adverse Events, EOT: end of treatment, w: weeks.
